# Association of antithrombin with development of trauma-induced disseminated intravascular coagulation and outcomes

**DOI:** 10.3389/fimmu.2022.1026163

**Published:** 2022-12-09

**Authors:** Takeshi Wada, Atsushi Shiraishi, Satoshi Gando, Daijiro Kabata, Kazuma Yamakawa, Seitaro Fujishima, Daizoh Saitoh, Shigeki Kushimoto, Hiroshi Ogura, Toshikazu Abe, Toshihiko Mayumi, Yasuhiro Otomo

**Affiliations:** ^1^ Division of Acute and Critical Care Medicine, Department of Anesthesiology and Critical Care Medicine, Hokkaido University Faculty of Medicine, Sapporo, Japan; ^2^ Emergency and Trauma Center, Kameda Medical Center, Kamogawa, Japan; ^3^ Department of Acute and Critical Care Center, Sapporo Higashi Tokushukai Hospital, Sapporo, Japan; ^4^ Department of Medical Statistics, Graduate School of Medicine, Osaka Metropolitan University, Osaka, Japan; ^5^ Department of Emergency Medicine, Osaka Medical and Pharmaceutical University, Takatsuki, Japan; ^6^ Center for General Medicine Education, Keio University School of Medicine, Tokyo, Japan; ^7^ Division of Traumatology, Research Institute, National Defense Medical College, Tokorozawa, Japan; ^8^ Division of Emergency and Critical Care Medicine, Tohoku University Graduate School of Medicine, Sendai, Japan; ^9^ Department of Traumatology and Acute Critical Medicine, Osaka University Graduate School of Medicine, Suita, Japan; ^10^ Department of Emergency and Critical Care Medicine, Tsukuba Memorial Hospital, Tsukuba, Japan; ^11^ Health Services Research and Development Center, University of Tsukuba, Tsukuba, Japan; ^12^ Department of Trauma, Critical Care Medicine, and Burn Center, Japan Community Healthcare Organization, Chukyo Hospital, Nagoya, Japan; ^13^ Trauma and Acute Critical Care Center, Medical Hospital, Tokyo Medical and Dental University, Tokyo, Japan

**Keywords:** antithrombin, disseminated intravascular coagulation, innate immunity, soluble fibrin, thrombin, trauma-induced coagulopathy

## Abstract

**Introduction:**

Trauma activates the innate immune system to modulate hemostasis and minimize the damage caused by physiological bodily responses, including the activation of coagulation. Sufficiently severe trauma overwhelms physiological responses and elicits the systemic inflammatory response syndrome, which leads to the onset of disseminated intravascular coagulation (DIC), characterized by dysregulated inflammatory coagulofibrinolytic responses. Impaired anticoagulant mechanisms, including antithrombin, constitutes the pathology of DIC, while the dynamics of antithrombin and relevance to outcomes in trauma-induced coagulopathy have not been fully elucidated. This study investigated the associations of antithrombin activity with DIC onset and outcomes in severely injured patients.

**Methods:**

This retrospective sub-analysis of a multicenter, prospective study included patients with an injury severity score ≥16. We characterized trauma patients with low antithrombin activity (antithrombin <80% on hospital arrival, n = 75) in comparison with those who had normal antithrombin activity (antithrombin ≥80%, n = 200). Global markers of coagulation and fibrinolysis, molecular biomarkers for thrombin generation (soluble fibrin [SF]), and markers of anticoagulation (antithrombin) were evaluated to confirm the associations of antithrombin with DIC development and outcomes, including in-hospital mortality and the multiple organ dysfunction syndrome (MODS).

**Results:**

Patients with low antithrombin activity had higher prevalence of shock, transfusion requirements, and in-hospital mortality. Higher DIC scores and more severe organ dysfunction were observed in the low AT group compared to that in the normal AT group. Antithrombin activity on arrival at the hospital was an independent predictor of the development of DIC in trauma patients, and levels of SF increased with lower antithrombin values (antithrombin activity > 85%). Antithrombin activity at 3 h showed good predictive performance for in-hospital mortality, and a multivariable Cox proportional-hazard regression model with a cross-product term between the antithrombin and DIC showed that the in-hospital mortality in patients with DIC increased with decreased antithrombin activity. A multivariable logistic regression model showed that the odds for the development of MODS in patients with DIC increased with lower antithrombin values.

**Conclusion:**

Decreased antithrombin activity in trauma-induced coagulopathy is associated with poor outcomes through worsening of DIC.

## Introduction

Trauma-induced cellular/tissue injury activates the release of damage-associated molecular patterns (DAMPs), such as histones, mitochondrial DNA, nucleosomes, and high-mobility group box-1. DAMPs activate innate immunity to modulate hemostasis and minimize the damage *via* physiological bodily responses (1, 2). In case of severe trauma, this physiological process evolves into a pathologic response that manifests as the systemic inflammatory response syndrome (SIRS) (2, 3). SIRS elicits tissue factor expression on the surface of endothelial cells and monocytes, which leads to the activation of the extrinsic coagulation pathway (4). Furthermore, DAMPs induce neutrophil activation, followed by the release of neutrophil extracellular traps (NETs) that mainly comprise histones and promote further thrombin generation. These processes constitute the pathophysiology of disseminated intravascular coagulation (DIC), which is defined on the basis of dysregulated inflammatory coagulofibrinolytic responses to a traumatic insult (5, 6) and can induce poor outcomes through the development of consumption coagulopathy and multiple organ dysfunction syndrome (MODS) (7). Another important factor in the pathophysiology of DIC is impaired anticoagulant mechanisms (2). In the physiological hemostatic reaction, coagulation activation is controlled by the activation of anticoagulant pathways as a mechanism to prevent excessive thrombin generation. However, if an extremely severe reaction occurs, DAMP-induced inflammatory cytokines hamper anticoagulant pathways and thus lead to uncontrolled massive thrombin generation (8).

Antithrombin, which inhibits factor Xa and thrombin, is one of the important components of the natural anticoagulation system. Low antithrombin activity is observed in severely injured patients, especially in patients with DIC (9, 10), and potentially involves multifactorial causation, including antithrombin depletion through the formation of the thrombin–antithrombin complex, extravascular loss due to increased vascular permeability, and degradation by neutrophil elastase (11). Although decreased antithrombin activity is presumed to result in a deteriorated ability to localize hemostasis at the wound site and subsequent systemic thrombin generation, few studies have examined the role of antithrombin in the pathogenic mechanisms that underlie trauma-induced DIC.

Therefore, this study aimed to characterize trauma patients with low antithrombin activity and to investigate the associations of antithrombin with the onset and outcomes of DIC in severely injured patients.

## Materials and methods

### Study design, setting, and ethical approval

This retrospective study involved a sub-analysis of the data from a trauma cohort in the Japanese Association for Acute Medicine (JAAM) Focused Outcomes Research in Emergency Care in Acute Respiratory Distress Syndrome, Sepsis, and Trauma (FORECAST) Study, which validated the association of DIC in trauma-induced coagulopathy (5). The JAAM FORECAST study is a multicenter prospective study that enrolled acutely ill patients, including those with acute respiratory distress syndrome, sepsis, and trauma, and collected consecutive samples from 39 emergency departments (EDs) and intensive-care units (ICUs) in tertiary hospitals in Japan between April 1, 2016 and January 31, 2018. This study was approved under the condition that written informed consent was obtained from each patient or their next of kin by the JAAM and the Ethics Committee of all participating hospitals (JAAM, 2014-01; Hokkaido University Graduate School of Medicine, Head institute of the FORECAST group, 014-0307) and was performed in accordance with the tenets underlying the Declaration of Helsinki.

### Participants

The trauma cohort of the JAAM FORECAST study enrolled adult patients with severe trauma aged ≥ 16 years, with an Injury Severity Score (ISS) ≥16 and were recruited directly from the scene by the emergency services. The exclusion criteria were as follows: history of cardiac arrest and resuscitation in relation to the current trauma, receiving anticoagulants, history of hemorrhagic diathesis or coagulopathy, and transfer from other hospitals. The study’s sample size depended on the study period, and all participants were followed until discharge. The participants were divided into two groups: low antithrombin group and normal antithrombin group. The low- antithrombin group comprised patients with antithrombin activity < 80% immediately at hospital arrival (0 h) whereas the normal- antithrombin group comprised patients with antithrombin activity ≥80% at 0 h, based on the previous study of antithrombin in trauma (12) which specified a cutoff at 80% antithrombin activity and according to studies that reports a normal range of antithrombin activity of 80–130% (13, 14). Moreover, 27 healthy volunteers, unmatched for age and sex, were enrolled to evaluate the control values of the measured markers.

### Definition and diagnosis

DIC was diagnosed in accordance with the JAAM DIC diagnostic criteria (15) (**Supplementary Table 1**), which have been repeatedly validated for use in trauma cases (5, 9, 16). The prothrombin time International Normalized Ratio (INR) was used instead of the prothrombin time ratio for the diagnosis of DIC. The DIC scores were evaluated at 0, 3, and 24 h, and DIC was diagnosed if the DIC criteria were met at least once during the study period. Organ dysfunction was assessed based on the Sequential Organ Failure Assessment (SOFA) score. Furthermore, the SOFA score, without the coagulation score, was calculated to avoid an overlapping of platelet counts in both the DIC and SOFA scores. Each SOFA score ≥2 was considered a dysfunction of the corresponding organ, whereas MODS was defined as two or more organ dysfunctions without a coagulative dysfunction. Massive transfusion was defined as the transfusion of packed red blood cells of more than the estimated circulating blood volume (7.5% of body weight) within 24 h after presentation to the ED. SIRS were used to assess systemic inflammation (17). A systolic blood pressure <90 mmHg at the scene or at the ED and lactate levels >2 mmol/L at the ED were defined as shock. The Charlson Comorbidity Index (CCI) was used to assess comorbidities (18).

### Data collection and measurements

We collected 15-mL blood samples from eligible patients in citrated tubes immediately after hospital arrival (0 h) and again at 3 h after admission (3 h). The blood samples were centrifuged at 4°C, and the plasma obtained was stored at −80°C in each hospital. The levels of the following coagulofibrinolytic molecular markers that were analyzed in the present study were measured at the central laboratory of the LSI Medience Corporation (Tokyo, Japan) and include: 1) soluble fibrin (SF) (a marker of direct thrombin generation; LA, IATRO SFII; LSI Medience); 2) antithrombin (a marker of anti-thrombin; chromogenic assay, HemosIL Antithrombin LQ; Instrumental Laboratory); 3) D-dimer (a marker of fibrinolysis; LPIA, LPIA GENESIS D-dimer; LSI Medience). The values of global coagulation and fibrinolysis markers, including PT-INR, activated partial thromboplastin time (APTT), fibrinogen, and fibrin/fibrinogen degradation products (FDP) were collected from the medical databases of each participating institution.

### Statistical analysis

The participants’ characteristics at hospital arrival (baseline) are reported as median with interquartile range for continuous variables and as the number with the proportion for categorical variables. The Mann–Whitney *U* and chi-square tests were used to determine the differences in the characteristics between the subgroups that were categorized according to the baseline antithrombin value. We conducted logistic regression analysis to evaluate the association between the development of DIC and the coagulofibrinolytic markers, including the antithrombin activity, at hospital arrival. Variables that were found to be statistically significant at the 10% level on univariate analysis were included subsequently in the multivariate analysis. Furthermore, to examine more complex clinical relationships, we performed multivariable nonlinear regression analyses to assess the associations between the level of soluble fibrin, which reflects the extent of thrombin generation – the main pathology in DIC – and the following clinical markers: PT-INR, APTT, fibrinogen, D-dimer, FDP, and antithrombin, with adjustment for the patients’ age and sex. The nonlinear association was evaluated by a restricted-cubic-spline function with three knots. Receiver operating characteristic (ROC) curve analyses with the area under the curve (AUC) calculation were used to quantify the predictive performance of the values of antithrombin activity at 0 and 3 h; the ΔAntithrombin, defined as the difference between antithrombin values at 0 and 3 h, was calculated to evaluate the importance of serial changes in antithrombin activity. The Youden Index was used to calculate the optimal cutoff point of the ROC curves. Furthermore, we examined the impact of the antithrombin level at 3 h after hospitalization on the patient’s outcomes. We utilized a multivariable Cox proportional-hazard regression model for estimating the effect on in-hospital mortality within 28 days. Simultaneously, a multivariable logistic regression model was used to evaluate the impact on the occurrence of MODS. In addition, we applied the multivariable regression models with a cross-product term between antithrombin and DIC to assess whether the effect of the antithrombin differed based on the presence or absence of the DIC. In these models, we considered the nonlinear effect of the antithrombin level and adjusted the effect of the patient’s age, sex, ISS, shock defined by lactate levels, the severity of head trauma, isolated traumatic brain injury, and CCI.

In all of the analyses described above, the missing values were multiply imputed *via* the predictive mean matching methods with five repetitions. The statistical hypothesis tests were conducted using a two-sided 5% significance level. SPSS version 26 (IBM Corp., Armonk, NY, USA) and R version 4.1.1 (https://cran.r-project.org/) were used for statistical analysis.

## Results

### Baseline characteristics of the participants

Among the 295 participants who were enrolled in the trauma cohort of the JAAM FORECAST study, we excluded 17 patients with missing data on the DIC score at 0 h, 1 patient with missing antithrombin values at 0 h, and 2 patients with an ISS <16. The remaining 275 eligible participants were divided into two groups: low AT (antithrombin activity <80% at 0 h, n = 75) and normal AT (antithrombin activity ≥80% at 0 h, n = 200) (**Supplementary Figure 1**).

The demographics of the participants are shown in **Table 1**. The low antithrombin group comprised a higher proportion of older adults and women than the normal antithrombin group. The prevalence of shock, transfusion requirements, and in-hospital mortality were higher in the low antithrombin group compared with the normal antithrombin group. In addition, higher DIC scores and more severe organ dysfunction were observed in the low antithrombin group compared with the normal antithrombin group.

**Table 1:** Demographics, parameters at the scene and admission to the emergency department, volume of transfusion, and mortality in the study groups.

	Low antithrombin group n=75	Normal antithrombin group n=200	*p*-value
Demographics			
Age (years)	72 (50–81)	55 (43–68)	<0.001
Male sex, n (%)	37 (49.3)	145 (72.5)	<0.001
Charlson Comorbidity Index	0 (0–1)	0 (0–0)	0.021
DIC 0 h, n (%)	48 (64.0)	73 (36.5)	<0.001
DIC 0 h score	4 (3–4)	3 (1–4)	<0.001
DIC 3 h, n (%)	59 (84.2)	66 (41.0)	<0.001
DIC 3 h, score	5 (4–7)	3 (2–4)	<0.001
DIC 24 h, n (%)	40 (62.5)	62 (36.2)	<0.001
DIC score 24 h	5 (3–6)	2 (1–4)	<0.001
Antithrombin 0 h	67 (57–74)	100 (89–109)	<0.001
Antithrombin 3 h	72 (58–80)	96 (85–107)	<0.001
ISS	29 (25–35)	26 (20–29)	<0.001
AIS			
Head	3 (0–4)	3 (0–4)	0.589
Face	0 (0–0)	0(0–1)	0.217
Neck	0 (0–0)	0 (0–0)	0.486
Thorax	3 (0–4)	3(0–4)	0.184
Abdomen	0 (0–2)	0 (0–0)	0.111
Spine			
Cervical	0 (0–0)	0 (0–0)	0.916
Thoracic	0 (0–0)	0 (0–0)	0.855
Lumbar	0 (0–0)	0 (0–0)	0.710
Upper extremity	0 (0–2)	0(0–2)	0.912
Lower extremity	0(2–4)	0(0–2)	<0.001
External	0 (0–1)	0(0–1)	0.068
SIRS criteria	2 (1–3)	2 (1–3)	0.165
Total SOFA score at 24 h after admission	7 (5–9)	4 (3–7)	<0.001
Central nervous system	3 (0–4)	1 (0–3)	0.001
Cardiovascular system	1 (0–1)	0 (0–1)	0.001
Respiratory system	2 (1–2)	1 (1–2)	0.014
Coagulation system	1 (0–2)	1 (0–1)	<0.001
Liver function	0 (0–1)	0 (0–1)	0.136
Renal function	0 (0–0)	0 (0–0)	0.002
SOFA score without coagulation	6 (4–8)	3 (2–5)	<0.001
MODS 24 h after admission	29 (50.0)	37 (29.8)	0.007
Number of organ dysfunctions 24 h after admission	1 (1–2)	1 (0–1)	0.001
Shock at the scene, n (%)	24 (32.0)	31 (15.6)	0.003
Shock at ED, n (%)	29 (38.7)	18 (9.0)	<0.001
Massive transfusion, n (%)	33 (45.2)	19 (9.7)	<0.001
Tranexamic acid, n (%)	37 (49.3)	98 (49.0)	0.534
Operation within 24 h after admission, n (%)	55 (74.3)	94 (48.5)	<0.001
At the scene			
Systolic blood pressure (mmHg)	100 (82–133)	123 (96–144)	0.003
Diastolic blood pressure (mmHg)	66 (49–74)	76 (59–97)	0.006
Heart rate (beats/min)	93 (80–114)	84 (72–102)	0.001
Respiratory rate (breaths/min)	20 (18–24)	24 (18–28)	0.792
At the ED			
Systolic blood pressure (mmHg)	103 (78–132)	137 (108–155)	<0.001
Diastolic blood pressure (mmHg)	62 (52–76)	83 (65–100)	<0.001
Heart rate (beats/min)	90 (74–100)	87 (70–102)	0.001
Respiratory rate (breaths/min)	21 (18–26)	21 (18–25)	0.836
Lactate level (mmol/L)	2.3 (1.6–3.9)	2.4 (1.7–3.7)	0.013
Body temperature (°C)	36.5 (35.7–36.8)	36.6 (36.0–37.0)	0.750
3 h-transfusion requirements			
Packed red blood cells (mL)	280 (0–840)	0 (0–0)	<0.001
Fresh frozen plasma (mL)	480 (0–720)	0 (0–0)	<0.001
Platelet concentrate (U)	0 (0–0)	0 (0–0)	0.001
24 h-transfusion requirements			
Packed red blood cells (mL)	1260 (560–3920)	0 (0–560)	<0.001
Fresh frozen plasma (mL)	1440 (0–2880)	0 (0–480)	<0.001
Platelet concentrate (U)	0 (0–0)	0 (0–0)	<0.001
In-hospital mortality, n (%)	13 (17.8)	16 (8.0)	0.021

Reported proportions (counts) for categorical variables and medians (interquartile ranges) for continuous variables. Shock was defined based on a systolic blood pressure <90 mmHg.

AIS, Abbreviated Injury Scale; DIC, disseminated intravascular coagulation; ED, emergency department; ISS, Injury Severity Score; MODS, multiple organ dysfunction syndrome; SIRS, systemic inflammatory response syndrome; SOFA, Sequential Organ Failure Assessment.

### Factors associated with DIC development in trauma patients

**Figure 1:**
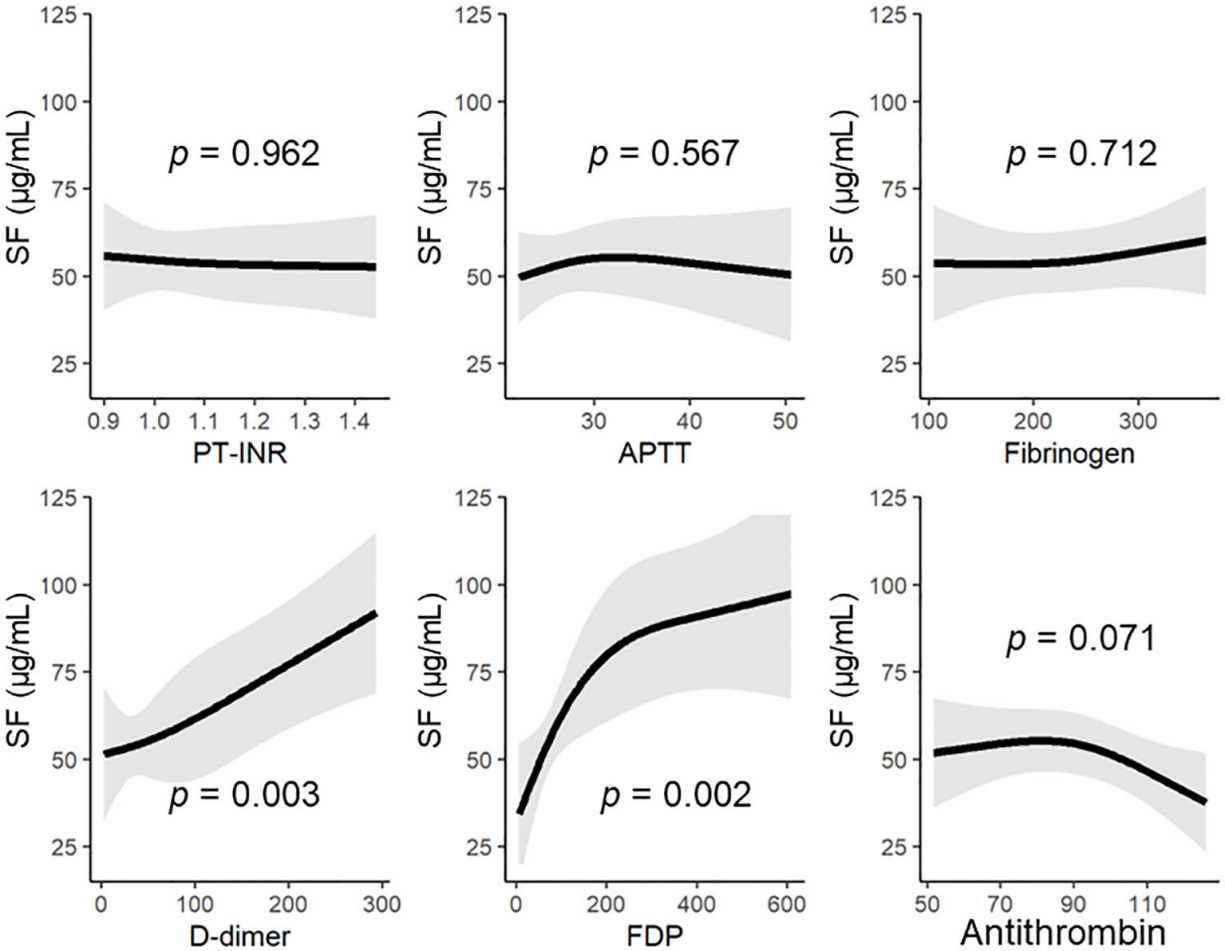
The SF levels on hospital arrival based on the general coagulofibrinolytic markers, including antithrombin. Regression lines of the levels of SF were estimated by multivariable nonlinear regression analyses, with adjustment for the patients’ age and sex. The nonlinear association was allowed by a restricted-cubic-spline function with three knots. APTT, activated partial thromboplastin time; AT, antithrombin; FDP, fibrin/fibrinogen degradation products; PT-INR, prothrombin time International Normalized Ratio; SF, soluble fibrin.

Multiple logistic regression analysis showed that the levels of D-dimer and antithrombin at 0 h were independent predictors of DIC development (**Table 2**). Serial changes in the values of molecular markers, including antithrombin, SF, and D-dimer, in patients with and without DIC are also shown in **Supplementary Figure 2**. Furthermore, we evaluated the correlations of SF—which reflects thrombin generation, the main pathology in DIC—to general coagulofibrinolytic markers by using a multivariable nonlinear regression analysis based on a restricted-cubic-spline method (**Figure 1**). In general, the SF values were constant, regardless of the values of PT-INR, APTT, and fibrinogen; thus, there were no significant correlations between the SF values and these markers. In contrast, the values of D-dimer and FDP significantly correlated with the SF values (*p* = 0.003 and 0.002, respectively) and that of antithrombin tended to correlate with the SF values (*p* = 0.071). The SF values increased with lower antithrombin values (antithrombin activity > approximately 85%).

**Table 2:** Logistic regression analysis of variables that predict DIC onset during the 24 h after admission.

Variables	Univariate		Multivariate	
	OR (95% CI)	*p*-value	HR (95% CI)	*p*-value
Age, years	1.004 (0.990–1.018)	0.595		
Sex	0.510 (0.273–0.954)	0.035		
Charlson Comorbidity Index	1.293 (0.840–1.988)	0.243		
Platelet count at 0 h	0.999 (0.996–1.002)	0.999		
PT–INR 0 h (OR per 0.01 Unit)	1.056 (1.026–1.086)	<0.001		
APTT 0 h	1.110 (1.043–1.181)	0.001		
Antithrombin 0 h	0.691 (0.945–0.997)	<0.001	0.967 (0.943–0.991)	0.007
Fibrinogen 0 h	0.992 (0.988–0.996)	<0.001		
FDP 0 h	1.033 (1.021–1.046)	<0.001		
D–dimer 0 h	1.071 (1.047–1.095)	<0.001	1.081 (1.050–1.112)	<0.001

APTT, activated partial thromboplastin time; FDP, fibrin/fibrinogen degradation products; PT-INR, prothrombin time International Normalized Ratio.

### Association of antithrombin with outcomes

**Figure 2:**
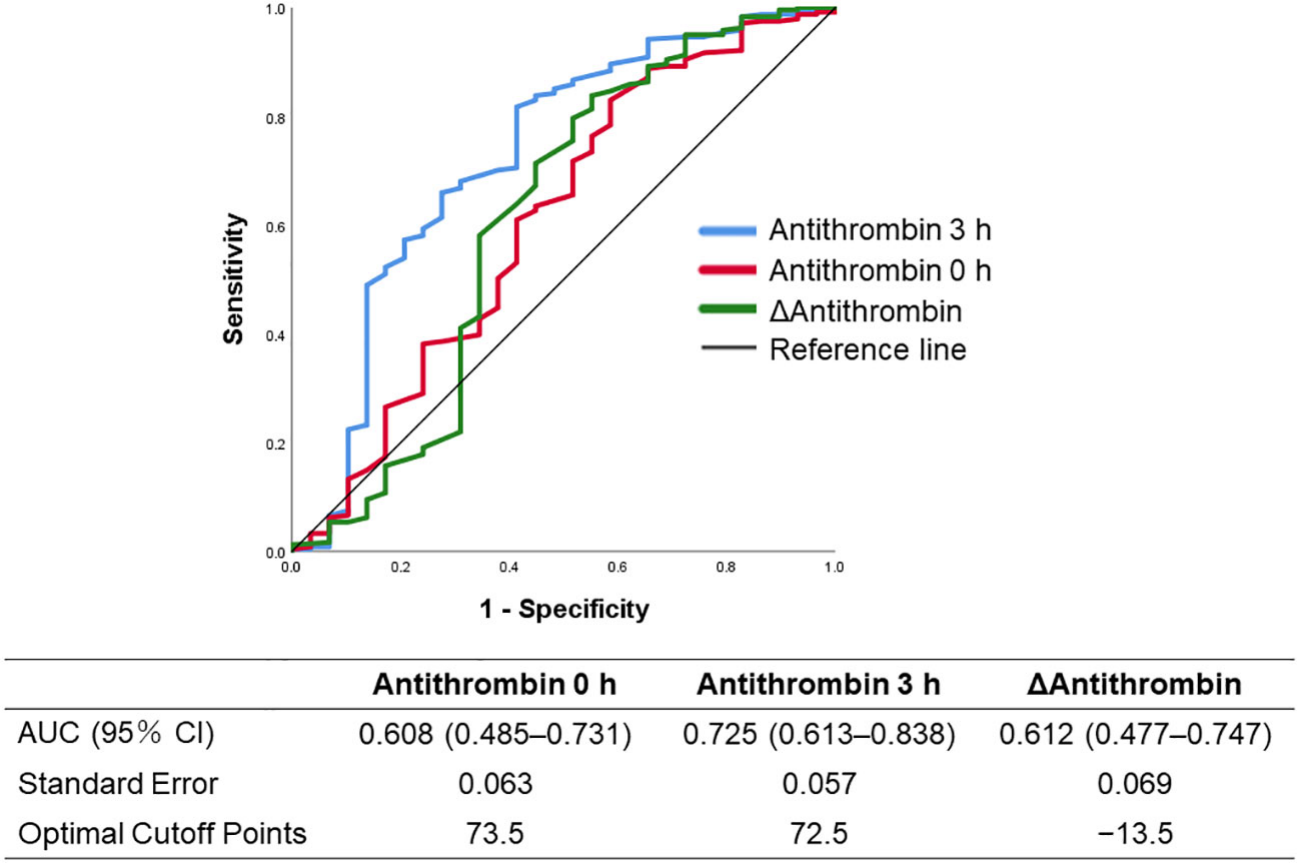
Receiver operating characteristics curves of the antithrombin-related values (antithrombin values at 0 and 3 h, and Δantithrombin) to predict in-hospital mortality. Δantithrombin was defined as the difference between the antithrombin values at 0 and 3 h. AUC, area under the curve.

**Figure 3:**
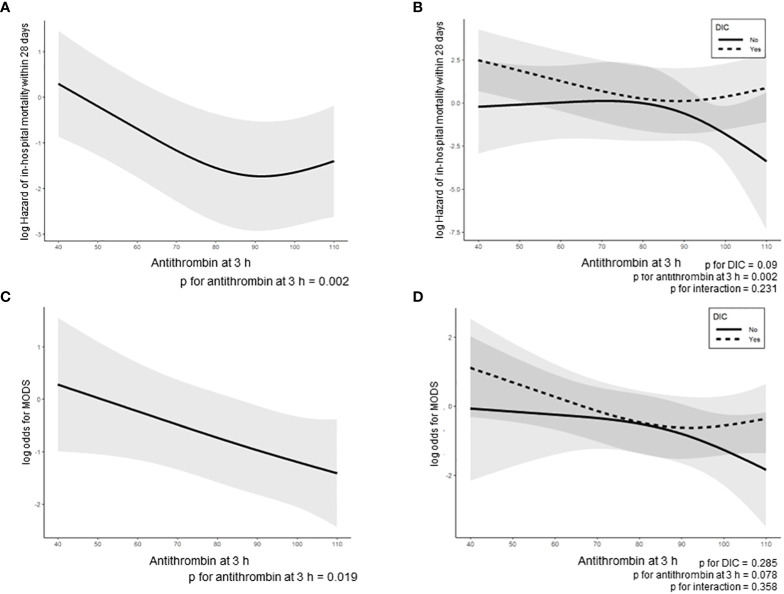
Regression line of in-hospital mortality in all enrolled trauma patients was dependent on antithrombin activity at 3 h, as estimated by the Cox proportional hazard regression model **(A, B)** and a two-way interaction term between the antithrombin activity at 3 h and the presence or absence of DIC onset during the 24 h after hospital arrival **(B)**. Regression line of the development of MODS during the 24 h after admission based on the multivariable logistic regression model **(C, D)** and a two-way interaction term between the antithrombin activity at 3 h and the presence or absence of DIC onset during the 24 h after hospital arrival **(D)**. The lines indicate estimated log-transformed relative hazards or odds, and the shaded areas represent 95% confidence intervals. In 3B and 3D, dotted and solid lines represent the patients with and without DIC, respectively. DIC, disseminated intravascular coagulation.

The ROC curves of the antithrombin levels at 0 and 3 h as well as the ΔAntithrombin in the prediction of in-hospital mortality are shown in **Figure 2**. Among these three antithrombin-related values, the antithrombin activity at 3 h had the best predictive performance for in-hospital mortality (AUC 0.725, SE 0.057). The optimal cutoff point, which was calculated using the Youden Index, was 72.5%. Based on this result, multivariate Cox proportional hazard regression analyses were performed to evaluate the outcomes, including in-hospital mortality and the development of MODS, which were dependent on antithrombin value at 3 h. The in-hospital mortality risk of all patients increased with a decrease in the antithrombin values (antithrombin activity < approximately 90%) (**Figure 3A**). Especially in trauma patients with DIC, the risk increased with lower antithrombin values (antithrombin activity < approximately 80%) (**Figure 3B**). Multiple logistic regression analysis showed similar results in that the odds for the development of MODS increased according to lower value of antithrombin (**Figure 3C**). Furthermore, the odds for the development of MODS in patients with DIC increased with a lower antithrombin value (antithrombin activity < approximately 80%) (**Figure 3D**).

## Discussion

We investigated the associations of antithrombin with the development of DIC and the outcomes in patients with severe trauma to gain a novel insight into the role of antithrombin in the pathophysiology of trauma-induced coagulopathy. Patient group with low antithrombin activity had a higher proportion of elderly individuals and women, more severe injury, higher DIC scores, more severe organ dysfunction, and a higher mortality risk than the group with normal antithrombin activity. The antithrombin activity on arrival at the hospital was an independent predictor of the development of DIC in trauma patients, and levels of SF – a marker of thrombin generation – increased with lower antithrombin values (antithrombin activity > 85%). Moreover, antithrombin activity at 3 h showed a good predictive performance regarding in-hospital mortality, which increased in patients with DIC and lower antithrombin activity.

Two conflicting theories—regarding DIC (2, 19) and acute traumatic coagulopathy (20, 21), which posits that the main pathophysiologies of trauma-induced coagulopathy (TIC) are activated protein C-mediated suppression of coagulation—have been advocated over the past decade regarding the primary pathogenesis of TIC. In 2020, a consensus statement from the International Society on Thrombosis and Haemostasis defined TIC with perspectives of these two theories (22). However, this new definition of TIC makes little mention of antithrombin, a crucial coagulation control mechanism. To gain a more detailed understanding of the pathogenesis of TIC, this study examined the dynamics and clinical values of antithrombin in patients with severe trauma.

In physiological conditions, the activation of coagulation as a result of the normal response to insults is controlled by anticoagulant-related mechanisms that involve protease inhibitor and protein C, which inhibit excessive thrombin generation. Plasma serine protease inhibitors (SERPIN) plays a central role in controlling coagulation activation by protease inhibitors. Among the SERPIN, antithrombin has the broadest inhibitory activity against coagulation-associated serine proteases and is the most important inhibitor of thrombin (23). In pathological reactions due to SIRS, which is caused by sufficiently severe insults, the decreased antithrombin activity—which occurs due to antithrombin consumption for the neutralization of thrombin and extravascular leakage due to increased vascular permeability—further enhances thrombin generation, which constitutes the thrombin burst—an essential component in the pathophysiology of DIC (11). A recent study has referred to DIC as “dysregulated inflammatory coagulofibrinolytic responses,” wherein NETs and histones act synergistically (5, 6, 24). The degradation of antithrombin by NET-associated elastase reduced synthesis in liver owing to histone-induced liver injury and loss to the endovascular space due to histone-induced increased vascular permeability are the major mechanisms that lead to low antithrombin activity (24). Furthermore, the fact that antithrombin administration improved survival in a mouse model of endotoxemia through the attenuation of NETs supports the potential of antithrombin as therapy for DIC (25).

**Table 1** shows that the low AT group had a higher proportion of elderly individuals and women. Previous studies have suggested that hypercoagulability in women after injury may be caused by the effects of estrogen (7, 26, 27). In addition, increased tissue factor expression, which activates extrinsic coagulation pathway, has been found in elderly patients (28). These evidences support that age- and sex-related hypercoagulability after trauma lead to increased thrombin generation, which is caused by consumptive decrease in antithrombin activity due to the formation of thrombin-antithrombin complex. However, age and sex were not extracted as independent predictors of DIC development by multivariate logistic regression analysis (**Table 2**), suggesting that age and sex may not have a significant effect on the development of pathological coagulofibrinolytic responses.

The present study showed that antithrombin activity at 0 h was an independent predictor of DIC onset within 24 h after admission (**Table 2**). Similar results in patients with sepsis were reported from a previous study which showed that the antithrombin level is a good indicator of DIC severity (29). In addition, Yanagida et al. demonstrated that antithrombin levels are an independent determinant of SF in trauma patients with DIC (30). Notably, **Figure 1** in the present study shows not only the correlation between antithrombin and SF but also the dynamics of SF in correlation with antithrombin levels, and that the SF values increased with lower antithrombin values (antithrombin activity > 85%). This result is supported by a previous *in vitro* study that assessed plasma samples obtained from trauma patients and showed that, when the plasma was diluted, thrombin generation progressively increased even if the antithrombin activity was within the normal range (31). Although a recent basic research from our laboratory demonstrated that thrombin generation significantly increased when antithrombin activity was ≤50% (32), the results of the present study suggest that higher antithrombin activity may be required to control thrombin generation under the condition of coagulation activation in trauma patients.

In clinical settings of sepsis, it has been repeatedly confirmed that low antithrombin activity is associated with poor survival outcome (33–36). To the best our knowledge, however, this is the first study to evaluate the association between antithrombin activity and survival outcome in trauma patients. The results show that, in addition to the baseline antithrombin activity, antithrombin activity after antithrombin supplementation and the difference between the pre- and post-antithrombin supplementation (ΔAntithrombin) levels are important predictors of mortality. Based on studies of antithrombin administration as anticoagulant therapy for sepsis-induced DIC (37, 38), we evaluated the predictive performance of antithrombin values at 0 and 3 h and the ΔAntithrombin for in-hospital mortality. Although the in-hospital mortality increased with lower antithrombin activity at 3 h, which had the best predictive performance for in-hospital mortality among the three antithrombin-related values (**Figure 3A**), this trend was found only in patients with DIC (**Figure 3B**). These results imply the importance of antithrombin in severe and pathological coagulofibrinolytic reactions to trauma and indicate that the correction of antithrombin activity may improve outcomes of severe trauma patients. Moreover, results shown in **Table 2** and **Figure 2** may indicate that the correction of antithrombin activity at 3 h after injury—determined by predicting the development of DIC using the initial AT values as an indicator—may contribute to improving the outcomes of trauma patients. Waydhas et al. evaluated the influence of antithrombin administration to achieve antithrombin activity of 140% in patients with multiple severe injuries on the outcomes, including mortality, and found no significant benefit of antithrombin administration (39). However, these results do not indicate that antithrombin administration in trauma patients has no therapeutic efficacy. The importance of correctly identifying an optimal patient population with sepsis that can benefit from anticoagulant therapy has been repeatedly reported (40–43); consequently, we suggest that for trauma patients it is necessary to clarify the type of bleeding (simple type hemorrhage or oozing type hemorrhage), the type of coagulation changes (DIC or not), and when antithrombin should be administered (acute phase or sub-acute phase) to effectively identify target patients that can benefit from antithrombin administration. The results of this study provide important evidence that forms the basis for undertaking randomized controlled trials to test the efficacy of antithrombin correction in trauma-induced DIC.

Several limitations of our study need to be considered. First, although this study comprised a sub-analysis of data from a prospectively conducted study, causal relationships could not be demonstrated because of the retrospective study design. Second, antithrombin is widely known to have anti-inflammatory properties in addition to its anticoagulant effects, which was not evaluated in this study. Third, we did not distinguish the types of trauma. In particular, it has been noted that traumatic brain injury (TBI) may induce a specific immune response. However, our previous study demonstrated similar coagulofibrinolytic changes in isolated TBI and non-TBI trauma patients when the ISS was comparable (44). Fourth, since some patients enrolled in this study received transfusion therapy such as fresh frozen plasma (28.1%), platelet (5.3%), and cryoprecipitate (0.4%) within 3 h after hospital arrival, these transfusions may have affected the antithrombin activity and the DIC scores at 3 h. Fifth, in the low antithrombin group, there was a higher proportion of lower extremity injuries (**Table 1**). Because lower extremities in Abbreviated Injury Scale coding include pelvic fractures, which are often severe and cause massive hemorrhage, we postulate that this may be one of the reasons of the lower AT activity, implying a selection bias of the patient cohort in this study. Finally, as this study was conducted in a single industrialized country, the results may lack generalizability.

## Conclusions

The present study demonstrated that the antithrombin activity immediately after trauma was strongly associated with the development of DIC in severely injured patients. Furthermore, antithrombin activity at 3 h after hospital arrival predicted the in-hospital mortality in trauma patients with DIC. These results support the importance of antithrombin in pathological coagulofibrinolytic responses after severe trauma. Multinational studies with more diverse population are needed to confirm the findings of this study. Thus, this study serves as a catalyst for producing evidence wherein interventions that target antithrombin activity can be used to improve outcomes in severe trauma patients.

## Data availability statement

The original contributions presented in the study are included in the article/**Supplementary Material**. Further inquiries can be directed to the corresponding author.

## Ethics statement

The studies involving human participants were reviewed and approved by The Japanese Association for Acute Medicine and the Ethics Committee of Hokkaido University Graduate School of Medicine. The patients/participants provided their written informed consent to participate in this study.

## Member of the JAAM FORECAST Study group

We wish to express our gratitude to the JAAM FORECAST Study group:

Keio University School of Medicine (Junichi Sasaki), Kobe University Graduate School of Medicine (Joji Kotani), Aichi Medical University Hospital (Naoshi Takeyama), Yamaguchi University Hospital (Ryosuke Tsuruta), Kawasaki Municipal Hospital (Kiyotsugu Takuma); Kurume University (Norio Yamashita), Aizu Chuo Hospital (Shin-ichiro Shiraishi), Teikyo University School of Medicine (Hiroto Ikeda), Kawasaki Medical School (Yasukazu Shiino), Kyorin University School of Medicine (Takehiko Tarui), Chiba University Graduate School of Medicine (Takaaki Nakada), St. Luke’s International Hospital (Toru Hifumi), Kitakyushu City Yahata Hospital (Kohji Okamoto), Saga University Hospital (Yuichiro Sakamoto), Center Hospital of the National Center for Global Health and Medicine (Akiyoshi Hagiwara), Nippon Medical School (Tomohiko Masuno), Community Healthcare Organization, Chukyo Hospital (Masashi Ueyama), Osaka General Medical Center (Satoshi Fujimi, Yutaka Umemura), and the JAAM FORECAST Trauma investigators: Osaka City University Hospital (Yasumitsu Mizobata); National Hospital Organization Sendai Medical Center (Yasuo Yamada); Saitama Medical University Saitama Medical Center (Satoru Sugiyama); Fukui Prefectural Hospital (Hiroshi Ishida); Sapporo Medical University (Eichi Narimatsu); Fukuyama City Hospital (Koji Miyasho); Hiratsuka City Hospital (Toshio Kanai); Saiseikai Utsunomiya Hospital (Satoru Miyatake); Japanese Red Cross Society Kyoto Daini Hospital (Ryouji Iiduka); Shinsyu University School of Medicine (Hiroshi Imamura); Rinku General Medical Center (Yasuaki Mizushima); Subaru Health Insurance Society Ota Memorial Hospital (Yoshitake Sato); Saitama Medical University International Medical Center (Manabu Nemoto); Aomori Prefectural Central Hospital (Hiroyuki Hanada); National Hospital Organization Hokkaido Medical Center (Yasuo Shichinohe); Saga-ken Medical Centre Koseikan (Kenji Hirahara); Hachinohe City Hospital (Akihide Kon); Juntendo University Nerima Hospital (Manabu Sugita); Kawaguchi Municipal Medical Center (Yasutaka Naoe); Kakogawa West City Hospital (Manabu Kirita); Osaka National Hospital (Daikai Sadamitsu); Kouseiren Takaoka Hospital (Masahiro Yoshida). Additionally, the JAAM FORECST Study Group thanks Shuta Fukuda for his special assistance in completing the study. This study was supported by the JAAM.

## Author contributions

TW analyzed the data, interpreted the results, and drafted the manuscript. AS verified the statistical methods and results. SG significantly influenced data interpretation and the critical appraisal of the manuscript. DK played a significant role in data analysis and the drafting of the manuscript. AS, SG, KY, SF, DS, SK, HG, TA, TM, and YO planned the study, decided the methodology, developed an online registration system, discussed the results, and critically revised the manuscript. All authors have read and approved the final version of the manuscript and agree to be accountable for the content of the work.
