# Supplementary material for: Association of antithrombin with development of trauma-induced disseminated intravascular coagulation and outcomes
Source: Front Immunol. 2022 Dec 9;13:1026163. doi: 10.3389/fimmu.2022.1026163 (PMC9788110; doi:10.3389/fimmu.2022.1026163)

Supplementary Material

**Supplementary Table 1. Scoring system for disseminated intravascular coagulation (DIC) by the Japanese Association for Acute Medicine (JAAM)**

----------------------------------------------------------------------------------------------------------------

1. Clinical conditions that may be associated with DIC

1) Sepsis/severe infection (any micro-organism)

2) Trauma/burn/surgery

3) Vascular abnormalities

- large vascular aneurysms

- giant hemangioma

- vasculitis

4) Severe toxic or immunological reactions

- snakebite

- recreational drugs

- transfusion reactions

- transplant rejection

5) Malignancy (except bone marrow suppression)

6) Obstetric calamities

7) Conditions that may be associated with SIRS

- organ destruction (e.g. severe pancreatitis)

- severe hepatic failure

- ischemia/hypoxia/shock

- heat stroke/malignant syndrome

- fat embolism

- rhabdomyolysis

- other

8) Other

-----------------------------------------------------------------------------------------------------------

2. Clinical conditions that should be carefully ruled out

A. Thrombocytopenia

1) Dilution and abnormal distribution

Massive blood loss and transfusion, massive infusion

2) Increased platelet destruction

ITP, TTP/HUS, HIT, drugs, viral infection, alloimmune destruction, APS, HELLP, extracorporeal circulation

3) Decreased platelet production

Viral infection, drugs, radiation, nutritional deficiency (vitamin B12, folic acid), disorders of hematopoiesis, liver disease, HPS

4) Spurious decrease

EDTA-dependent agglutinins, insufficient anticoagulation of blood samples

5) Other

Hypothermia, artificial devices in the vessel

B. Prolonged prothrombin time

Anticoagulation therapy, anticoagulant in blood samples, vitamin K deficiency, liver cirrhosis, massive blood loss, and transfusion

C. Elevated FDP

Thrombosis, hemostasis and wound healing, hematoma, pleural effusion, ascites, anticoagulant in blood samples, and antifibrinolytic therapy

D. Other

---------------------------------------------------------------------------------------------------------------

3. The diagnostic algorithm for SIRS

1) Temperature >38 ℃ or <36 ℃

2) Heart rate >90 beats/min

3) Respiratory rate >20 breaths/min or PaCO_2_ <32 torr (<4.3 kPa)

4) White blood cell >12,000 cells/mm^3^, <4,000 cells/mm^3^, or 10% immature (band) forms

---------------------------------------------------------------------------------------------------------------

4. The diagnostic algorithm

Score

SIRS criteria

≥3 1

0–2 0

Platelet counts (10^9^ /L)

<80 or >50% decrease within 24 h 3

≥80 to <120 or >30% decrease within 24 h 1

≥120 0

Prothrombin time (value of patient/normal value)

≥1.2 1

<1.2 0

Fibrin/fibrinogen degradation products (mg/L)

≥25 3

≥10 to <25 1

<10 0

Diagnosis

Four points or more DIC

-----------------------------------------------------------------------------------------------------------------

SIRS, systemic inflammatory response syndrome; ITP, idiopathic thrombocytopenic purpura; TTP, thrombotic thrombocytopenic purpura; HUS, hemolytic uremic syndrome; HIT, heparin-induced thrombocytopenia; APS, antiphospholipid syndrome; HELLP, hemolysis, elevated liver enzymes, and low platelet; HPS, hemophagocytic syndrome; EDTA, ethylenediaminetetraacetic acid; FDP, fibrin/fibrinogen degradation products.

**Supplementary Figure 1**. Flowchart of the selection of the study population


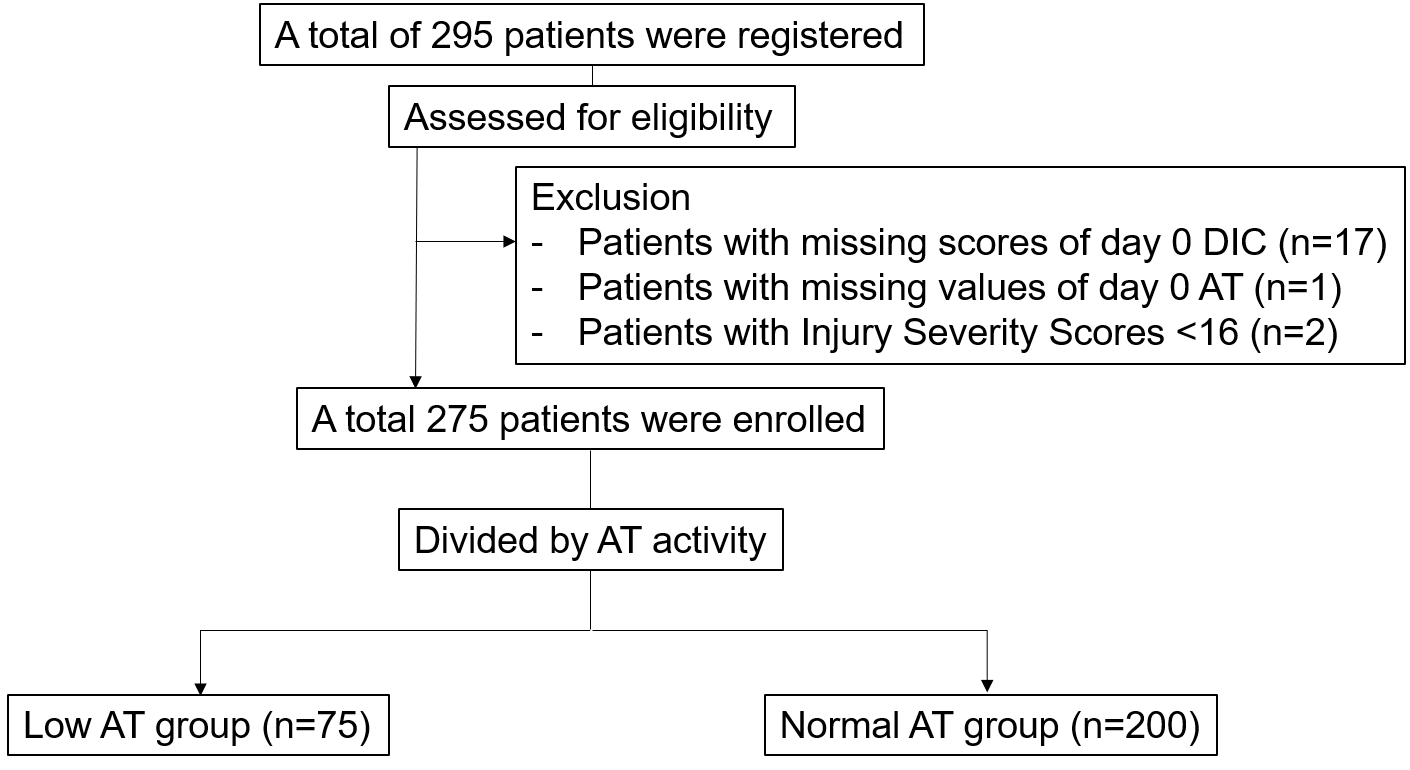


AT, antithrombin; DIC, disseminated intravascular coagulation

**Supplementary Figure 2**. Serial changes in the values of molecular markers, including antithrombin, soluble fibrin, and D-dimer, in patients with and without DIC


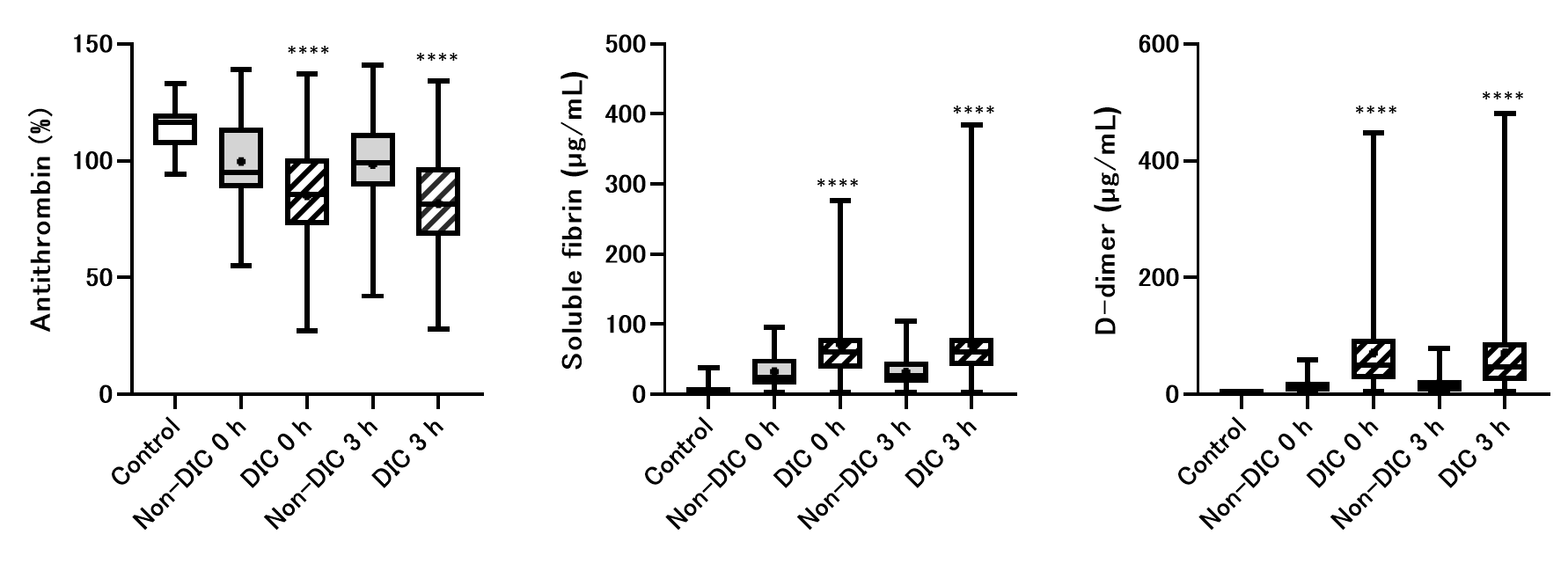


Antithrombin, soluble fibrin, and D-dimer values of healthy controls (white box), non-DIC (gray boxes), and DIC (hatched boxes) at presentation to the emergency department (0 h) and at 3 h after hospital arrival (3 h). Horizontal bars in the box indicate the median (middle) and interquartile ranges (upper 25% and lower 75%). Black boxes are mean values. **** *p*<0.0001 vs. non-DIC. DIC, disseminated intravascular coagulation.


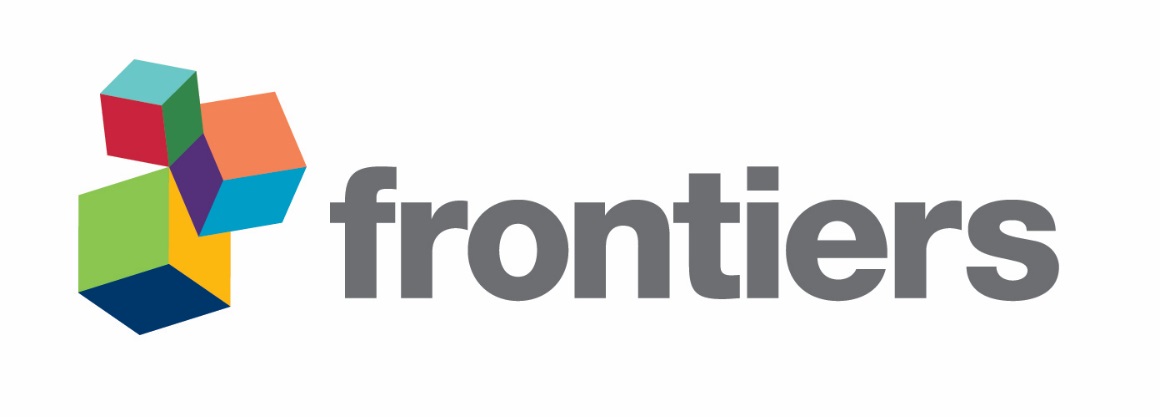

Supplement: Supplementary file 1 [file DataSheet_1.docx]
